# Complete Genome Sequence of a *Legionella longbeachae* Serogroup 1 Strain Isolated from a Patient with Legionnaires’ Disease

**DOI:** 10.1128/genomeA.00564-17

**Published:** 2017-06-15

**Authors:** Sandy Slow, Trevor Anderson, James Miller, Siddharth Singh, David Murdoch, Patrick J. Biggs

**Affiliations:** a Department of Pathology, University of Otago, Christchurch, New Zealand; b Microbiology, Canterbury Health Laboratories, Christchurch, New Zealand; c Millennium Science, Mulgrave, Victoria, Australia; d Pacific Biosciences, Menlo Park, California, USA; e Molecular Epidemiology and Veterinary Public Health Laboratory, Infectious Disease Research Centre, Institute of Veterinary, Animal and Biomedical Sciences, Massey University, Palmerston North, New Zealand

## Abstract

*Legionella longbeachae* serogroup 1, predominantly found in soil and composted plant material, causes the majority of cases of Legionnaires’ disease (LD) in New Zealand. Here, we report the complete genome sequence of an *L. longbeachae* serogroup 1 (sg1) isolate derived from a patient hospitalized with LD in Christchurch, New Zealand.

## GENOME ANNOUNCEMENT

*Legionella* spp. are intracellular bacterial pathogens that cause Legionnaires’ disease (LD), an often severe form of pneumonia. New Zealand (NZ) has the highest incidence of LD in the world (1), and *Legionella longbeachae* is the most clinically relevant species, particularly serogroup 1 (sg1) (2). It is predominantly found in soil and composted plant material (3, 4), and most cases occur over spring/summer, when the people at greatest risk are those involved in gardening activities (3, 5). Relative to *Legionella pneumophila*, the predominant disease-causing species in the United Kingdom, the United States, and Europe (1, 6), there are little genomic data for *L. longbeachae*. There is a complete genome and plasmid from an Australian sg1 isolate (NSW150; GenBank accession no. NC_013861.1), and recently, a second complete genome has become available for the ATCC type strain, which is the isolate obtained from the first reported *L. longbeachae* LD case from Long Beach, CA, USA, in 1981 (FDAARGOS_201; accession no. NZ_CP020412). Sequence analysis of the isolates has revealed a single circular chromosome of around 4.1 Mb, with an array of genes that contribute to its virulence and reflect its soil habitat (7–9). Here, we report the complete genome and plasmid sequences of an *L. longbeachae* sg1 clinical isolate obtained from a patient hospitalized with LD in 2014 from Christchurch, NZ.

The isolate was grown on buffered-charcoal-yeast-extract agar (72 h, 35°C), and DNA was purified using Genomic-tip 100/G (Qiagen, Hilden, Germany). Sequencing was conducted using the PacBio RSII (Menlo Park, CA, USA) and Illumina MiSeq (San Diego, CA, USA) systems. For RSII, one SMRTbell DNA library was constructed according to the 20-kb protocol and was size selected with BluePippin (15-kb cutoff). The library was sequenced using P6-C4 chemistry and a 240-min data collection time on one single-molecule real-time (SMRT) cell. The MiSeq library (250-bp paired-end) was prepared using the Nextera XT protocol and sequenced using version 2 chemistry. Approximately 53,000 RSII and 1,100,000 MiSeq reads were obtained for the isolate. The RSII data were assembled using the HGAP2 assembly pipeline in SMRT Analysis (version 2.3.0). A preassembly filter removed reads shorter than 500 bp or with a quality lower than 80%. The assembly was polished using Quiver, and the MiSeq reads were mapped onto the final RSII assembly using Pilon (version 1.20).

A single closed genome was constructed, consisting of a 4,162,768-bp chromosome and a 108,261-bp plasmid with G+C contents of 37.1% and 38.3%, respectively. Annotation was performed using the NCBI Prokaryotic Genome Annotation Pipeline (2013), which predicted 3,691 coding sequences for the chromosome and plasmid (3,577 and 114, respectively). There were 12 rRNAs, 52 tRNAs, and 4 noncoding RNAs (ncRNAs). Our chromosome and plasmid are 85.4 kb and 36.5 kb larger, respectively, than those of NSW150, while our chromosome is only 51 bp larger than that of FDAARGOS_201.

As whole-genome sequencing becomes commonplace in clinical diagnostics, understanding the genomic diversity of *Legionella* is important, especially for non-*pneumophila Legionella* species, for which data are sparse. Increasing the availability of *L. longbeachae* genomes may help establish the genetic relationships within and between species and help inform treatment and preventative measures.

### Accession number(s).

The PacBio and Illumina MiSeq sequence reads described here have been deposited at NCBI/GenBank under the BioProject number PRJNA369580. The whole-genome sequence described here has been deposited at NCBI/GenBank under the accession numbers CP020894.1 (chromosome) and CP020895.1 (plasmid).
